# Guidelines in review: Comparison of ESC and AHA guidance for the diagnosis and management of infective endocarditis in adults

**DOI:** 10.1007/s12350-018-1333-5

**Published:** 2018-06-19

**Authors:** David J. Murphy, Munaib Din, Fadi G. Hage, Eliana Reyes

**Affiliations:** 1 King’s College London and Guy’s and St Thomas’ NHS Foundation Trust PET Centre, London, UK; 2 Division of Cardiovascular Disease, Department of Medicine, The University of Alabama at Birmingham, Birmingham, AL USA; 3 Section of Cardiology, Birmingham Veteran Affairs Medical Center, Birmingham, AL USA; 4 Nuclear Medicine Department, Royal Brompton and Harefield NHS Foundation Trust, Sydney Street, London, SW3 6NP UK

**Keywords:** Infection, image-guided application, multimodality

## Abstract

Over recent years, new evidence has led a rethinking of the available guidance on the diagnosis and management of infective endocarditis (IE). This review compares the most recently available guidance provided by the American Heart Association (AHA) IE Writing Committee, and the Task Force for the management of IE of the European Society of Cardiology (ESC). This represents the sixth of a new series of comparative guidelines review published in the Journal.

Over recent years, new evidence has led to a rethink of the available guidance on the diagnosis and management of infective endocarditis (IE). This review compares the most recently available recommendations provided by the American Heart Association (AHA) IE Writing Committee, and the Task Force for the management of IE of the European Society of Cardiology (ESC).^1^,^2^ Class (I, II or III) and level of evidence (A, B or C) are provided for each recommendation where given by the guidelines (Tables 1, 2, 3; Figures 1, 2). As in previous comparative guidelines reviews published in the Journal,^3^–^7^ this review focuses on the role of imaging in the evaluation and management of patients with suspected IE.

**Table 1:**
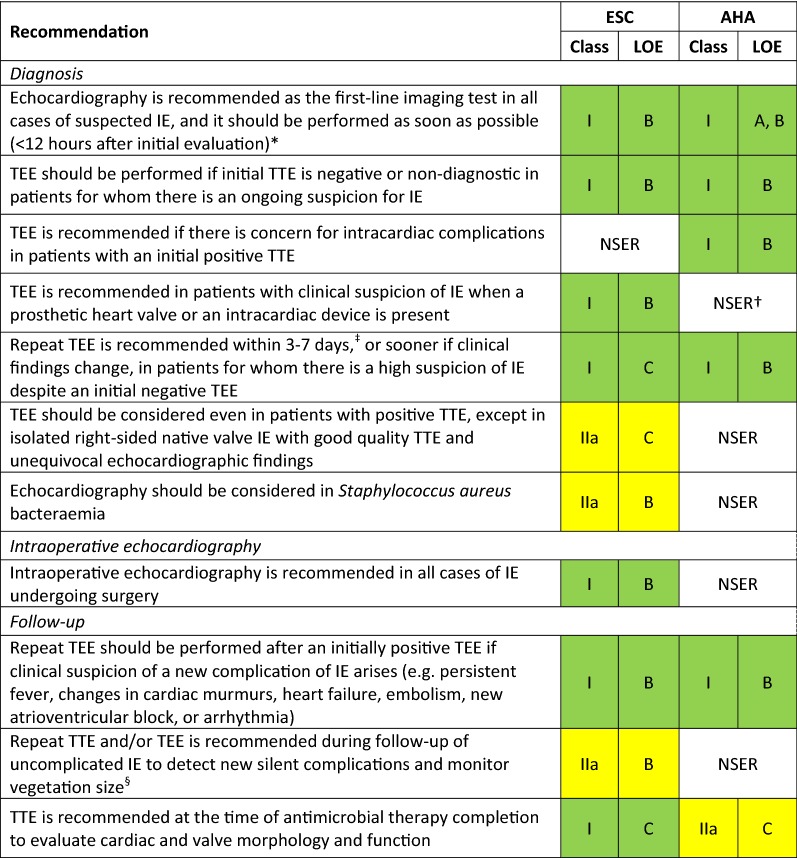
Indications for echocardiography in patients with suspected infective endocarditis

*According to the AHA scientific statement, TEE is preferred over TTE, but the latter should be performed if TEE is not immediately available. TTE may be sufficient in small children

^†^AHA statement also suggests TEE as first-line test in patients with a prosthetic valve and suspected IE

^‡^In this clinical scenario, the AHA statement recommends repeating the TEE in 3 to 5 days or sooner

^§^ESC guidelines stipulate that the timing and mode (TTE or TEE) of repeat test depend on initial findings, microorganism type, and initial response to therapy

**Table 2:**
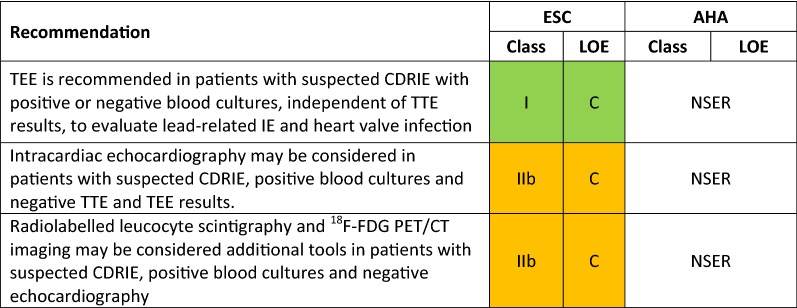
indications for non-invasive imaging in cardiac device-related infective endocarditis (CDREI)

**Table 3:**
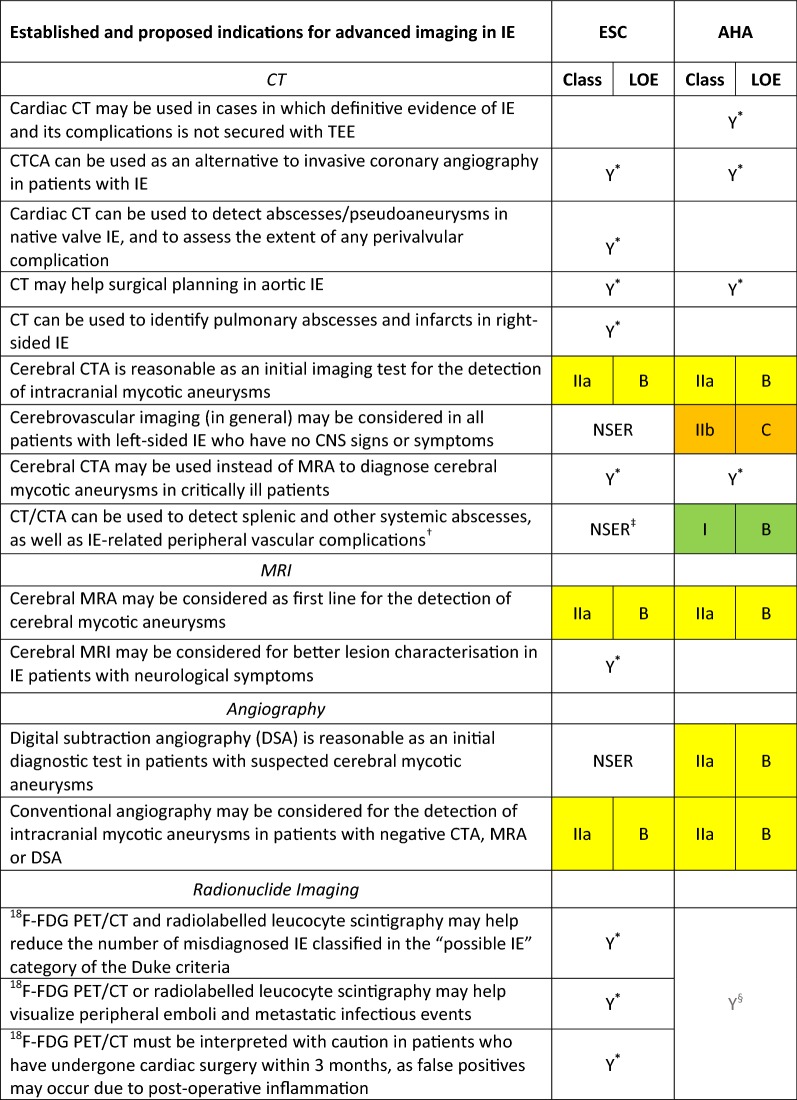
Role of CT, MRI, radionuclide imaging and angiography in the assessment of IE patients

*These proposed indications are discussed in the guidelines but neither the ESC guidelines nor the AHA scientific statement give specific or formal recommendation

^†^The AHA statement recommends that, in IE patients with suspected metastatic foci of infection, the choice of diagnostic technique (ultrasonography, CT or MRI) should be individualised for each patient (Class I; LOE, C)

^‡^Although there is no specific recommendation, the ESC guidelines state that patients with suspected splenic complications should be evaluated by CT, MRI or ultrasound

^§^The AHA statement recognises that more studies are needed to determine the role of ^18^F-FDG PET/CT imaging in the diagnosis and management of patients with IE, and highlights evidence on the usefulness of this technique for the detection of peripheral emboli and other extracardiac complications

**Figure 1:**
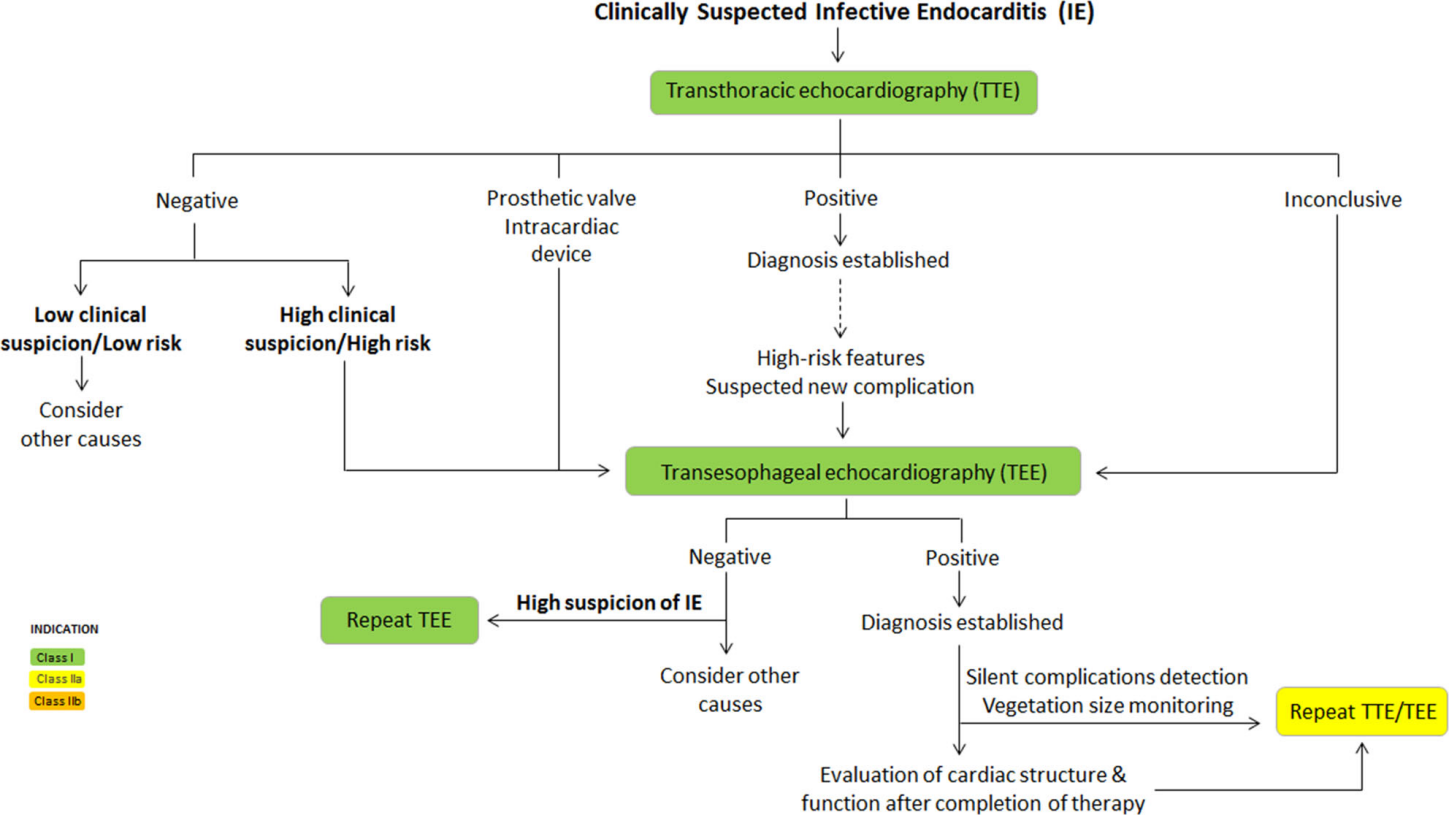
ESC and AHA recommendations for the initial assessment of patients with clinically suspected infective endocarditis using echocardiography

**Figure 2:**
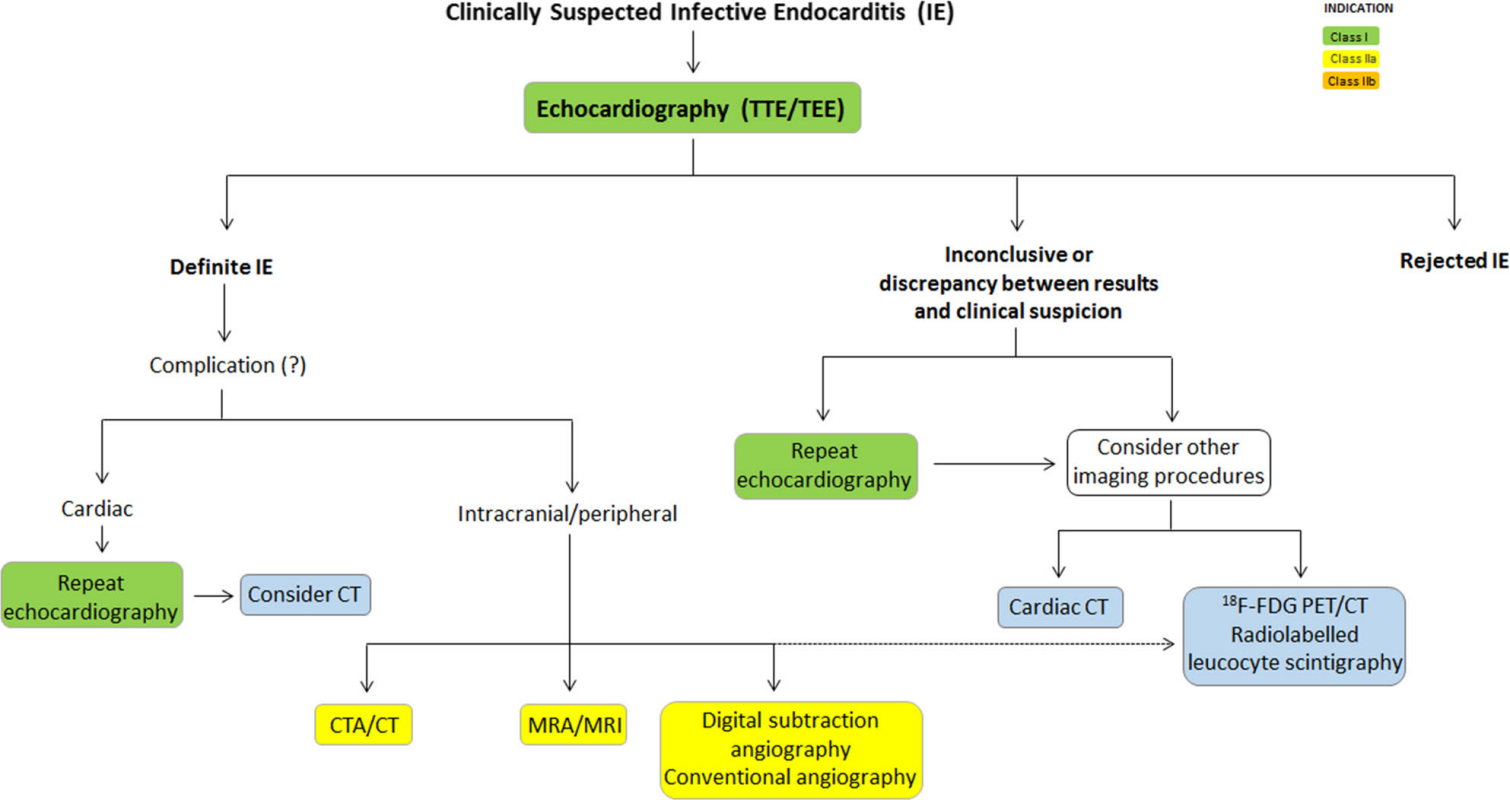
Role of advanced imaging in the assessment of patients with infective endocarditis. *CTA*, computed tomographic angiography; ^*18*^*F-FDG*, 18-fluorodeoxyglucose; *MRA*, magnetic resonance angiography; *MRI*, magnetic resonance imaging; *TEE*, transesophageal echocardiography; *TTE*, transthoracic echocardiography

## Abbreviations

CTCA: Computed tomographic coronary angiography
CDRIE: Cardiac device-related infective endocarditis
DSA: Digital subtraction angiography
^18^F-FDG: 18-fluorodeoxyglucose
IE: Infective endocarditis
LOE: Level of evidence
MRI: Magnetic resonance imaging
MRA: Magnetic resonance angiography
NSER: No specific equivalent recommendation
PET/CT: Positron emission tomography/computed tomography
TEE: Transesophageal echocardiography
TTE: Transthoracic echocardiography
